# Removing Auxetic Properties in f.c.c. Hard Sphere Crystals by Orthogonal Nanochannels with Hard Spheres of Another Diameter

**DOI:** 10.3390/ma15031134

**Published:** 2022-02-01

**Authors:** Jakub W. Narojczyk, Mikołaj Bilski, Joseph N. Grima, Przemysław Kędziora, Dmitrij Morozow, Mirosław Rucki, Krzysztof W. Wojciechowski

**Affiliations:** 1 Institute of Molecular Physics, Polish Academy of Sciences, M. Smoluchowskiego 17, 60-179 Poznan, Poland; kedziora@ifmpan.poznan.pl (P.K.); kww@ifmpan.poznan.pl (K.W.W.); 2 Institute of Applied Mechanics, Poznań University of Technology, Jana Pawla II 24, 60-965 Poznan, Poland; mikolaj.bilski@put.poznan.pl; 3 Department of Chemistry, Faculty of Science, University of Malta, MSD 2080 Msida, Malta; 4 Metamaterials Unit, Faculty of Science, University of Malta, MSD 2080 Msida, Malta; 5 Faculty of Mechanical Engineering, Kazimierz Pulaski University of Technology and Humanities in Radom, Stasieckiego 54, 26-600 Radom, Poland; d.morozow@uthrad.pl (D.M.); m.rucki@uthrad.pl (M.R.); 6 Akademia Kaliska im. Prezydenta Stanisława Wojciechowskiego, Nowy Świat 4, 62-800 Kalisz, Poland

**Keywords:** auxetics, negative Poisson’s ratio, nanolayers, hard sphere inclusions, Monte Carlo simulations

## Abstract

Negative Poisson’s ratio materials (called auxetics) reshape our centuries-long understanding of the elastic properties of materials. Their vast set of potential applications drives us to search for auxetic properties in real systems and to create new materials with those properties. One of the ways to achieve the latter is to modify the elastic properties of existing materials. Studying the impact of inclusions in a crystalline lattice on macroscopic elastic properties is one of such possibilities. This article presents computer studies of elastic properties of f.c.c. hard sphere crystals with structural modifications. The studies were performed with numerical methods, using Monte Carlo simulations. Inclusions take the form of periodic arrays of nanochannels filled by hard spheres of another diameter. The resulting system is made up of two types of particles that differ in size. Two different layouts of mutually orthogonal nanochannels are considered. It is shown that with careful choice of inclusions, not only can one impact elastic properties by eliminating auxetic properties while maintaining the effective cubic symmetry, but also one can control the anisotropy of the cubic system.

## 1. Introduction

Negative Poisson’s ratio (PR) [1] materials, or auxetics [2], as they are commonly referred to, are a relatively new class of materials exhibiting unusual elastic properties. The phenomena occurring inside their structure are responsible for their radically different deformation mechanisms during bending or stretching [3]. The ever growing interest in auxetics was sparked by the early theoretical [4,5,6,7] and experimental [8] studies performed in the 1980s. This interest is motivated by the vast potential applications [9,10,11,12] of materials that expand their transverse dimensions when stretched longitudinally (to point only one highly characteristic feature [13]). Since their discovery, auxetics have been extensively studied both theoretically [14,15,16,17,18] by computer simulations [19,20,21] and experimentally [22,23,24]. Auxetic properties, i.e., the existence of negative PR in at least some crystallographic directions (such materials are called partial auxetics [25]), were reported not only in model structures [13,26,27,28,29], but also in real cubic materials [30], polymers [31,32], composites [33], and foams [34,35]. Today, novel structures [36,37], nanostructures [38], and metamaterials [39,40,41] with auxetic properties are being developed. This would not have been possible without extensive theoretical studies in the form of basic research [42,43,44] or analysis and optimizations [45,46] of novel auxetic model structures and metamaterials. The study of materials with inclusions at the structural level is one of the possible directions of such optimizations [47,48,49,50,51].

Recently, inclusions in the form of periodic arrays of channels [49], layers [50], or their combination [51] on elastic properties of hard sphere face centered cubic (f.c.c.) crystal, have been investigated. The inclusions were formed by hard spheres with diameters different from the remaining particles of the crystal. It was shown that inclusions significantly impact the symmetry and elastic properties of the f.c.c. crystal. However, under the same thermodynamic conditions, different forms of inclusions exert extremely different effects on elastic properties. The inclusion in the form of an array of nanochannels oriented in [001]-direction filled by particles with larger diameters resulted in a significant enhancement of auxetic properties (e.g., PR in $\left[111\right]\left[11\bar{2}\right]$-direction decreased from 0.065 to −0.365, and the minimal value of PR decreased to −0.873 [49]), while the inclusion of similar particles forming nanolayers orthogonal to the [001]-direction showed only a slight enhancement of auxetic properties [50]. A surprising effect has been discovered while studying the effects of hybrid (joined) nanolayer and nanochannel inclusions in one system [51]. It was found that inclusion in such a form completely eliminated auxetic properties of the system. Since the negative value of PR is one of the characteristic features of most cubic systems [30], such a strong impact on the elastic properties of the f.c.c. crystal was not expected. In all three described cases, the introduced inclusions were also responsible for the change in symmetry from cubic to tetragonal one (the 422 symmetry class [52]). That research showed not only that adding the inclusions constitute a method for modifying the elastic properties of crystal systems, but more importantly that the shape and orientation of the inclusions play an important role in the final elastic properties, and also that the role is hard to predict. Thus, in this paper we study a triple inclusion in the form of nanochannels, but in different (mutually orthogonal) orientations.

Although this work is a purely theoretical research, there are techniques that potentially could be used to produce real systems similar to the models presented. One of such techniques is the ion implantation, a process that is widely used in many areas, e.g., semiconductor device fabrication or materials sciences in general [53,54]. It has recently been shown that implanting nitrogen ions into a cemented tungsten carbide guide pads for deep-hole drilling applications can substantially increase their hardness and durability [55]. These are also characteristic features of auxetic materials; however, the complicated mechanisms behind the changes of tribological, mechanical, and elastic properties of ion-implanted materials are not yet fully understood and explained. Thus, the lack of a theory describing these changes leaves the utility of this technique to produce auxetic materials an open question.

The structure of the paper is as follows: the most important aspects of the studied model are described in the following Section 2. In Section 3, essential information regarding elastic properties in the isobaric-isothermal ensemble are briefly described, and the details of computer simulations are provided. The results of the study and their discussion are placed in Section 4, followed by the last Section 5 that contains summary and conclusions.

## 2. The Model

The basis structure for the model, considered in this work, is the f.c.c. crystal of *N* hard spheres. Thus, the interaction between particles is of the form:

(1) $$\beta u_{ij}=\left\{\begin{array}{cc} \infty , & r_{ij}<\sigma_{ij},\\ 0, & r_{ij}\geq \sigma_{ij}. \end{array}\right.$$

where $r_{ij}$ is the distance between the centers of the interacting spheres *i* and *j*, $\sigma_{ij}=\left(\sigma_{i}+\sigma_{j}\right)/2$, with $\sigma_{i}$, $\sigma_{j}$ being the diameters of the respective spheres, $\beta =1/(k_{B}T)$, $k_{B}$ [J/K] is the Boltzmann constant, and *T* [K] is the temperature. Despite its simplicity the *hard sphere* (HS) potential is one of the fundamental interactions in liquid theory [56] and condensed matter physics [57], especially in regard to soft matter systems, e.g., liquid crystalline phases and colloids [57]. The HS system provides a very good insight into effects resulting from the relative particle dimensions. It constitutes the simplest approximation which includes short-range correlations originating from the excluded volume effects [57,58,59], and it is the simplest model that can exhibit melting. Moreover, the HS interaction allows one to mimic many of the properties of real systems, in this case most importantly, the existence of the negative PR [49,50,51,60].

The f.c.c. system, where all *N* particles have the same diameters equal to σ (which constitutes the unit of length), was modified by an arbitrary selection and replacement of the $N_{\mathrm{inc}}$ spheres with spheres with different diameters $\sigma^{\prime }\neq \sigma$. The clusters of replaced spheres are regarded as an inclusion implanted into the f.c.c. crystal (constituting the matrix for an inclusion). In this work, the inclusions are in the form of three nanochannels with mutually orthogonal layout. The concentration $c=N_{\mathrm{inc}}/N$ of the included particles depends on the selected size of the system $N=4N_{x}N_{y}N_{z}$ (where $N_{\alpha }$ are the numbers of unit cells of f.c.c. crystal in the respective directions), as well as on the diameter of the nanochannels and their layout in space.

**Figure 1:**
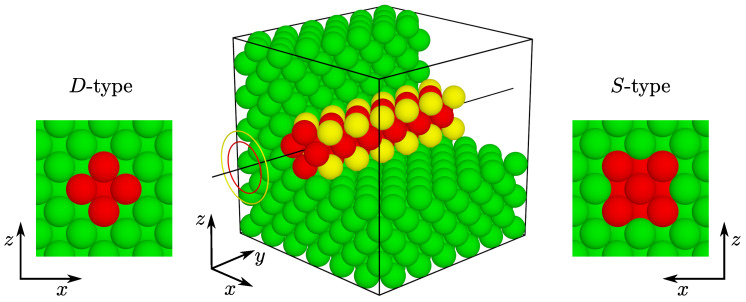
Illustration of two channels sizes studied in this work. Depending on the desired diameters (colored circles), the smaller channel incorporates the on-axis particles and their nearest neighbors (*D*-type), marked in red. The larger channel (*S*-type) contains all the *D*-type particles plus the particles from the second coordination zone around the channel axis, colored yellow. The yellow color has been used only to highlight the differences between the two channel sizes. The circles represent the diameters of the corresponding coordination zones. Inserts on the left and right present the *D*-type and *S*-type channels, respectively, viewed from the *y*-direction (along the channels’ axis). For clarity, part of the green (matrix) spheres located outside the channel have been removed from the image.

An array of nanochannels is introduced into the structure with designated orientation axis, its diameter, and a position in the model. In Figure 1, a single nanochannel is presented. The channel axis is oriented in the [010]-direction. The circles on the left illustrate the channel diameter. The inner circle (red) with diameter equal to 2σ, corresponds to the smaller channel, including particles placed on the axis and their nearest neighbors, also colored red. The outer (yellow) circle corresponds to the channel with diameter of $2\sqrt{2}\sigma$, containing all the red particles, plus the second nearest neighbors to the on-axis spheres, colored yellow. Due to the diamond-like and square-like shapes of the cross-section of the smaller and bigger nanochannels, we will refer to them as *D*-type and *S*-type, respectively, (as introduced in [61]). The diameter of the nanochannel can be arbitrarily increased to include particles in consecutive coordination zones; however, in this work we restrict ourselves to study only the two indicated sizes.

**Figure 2:**
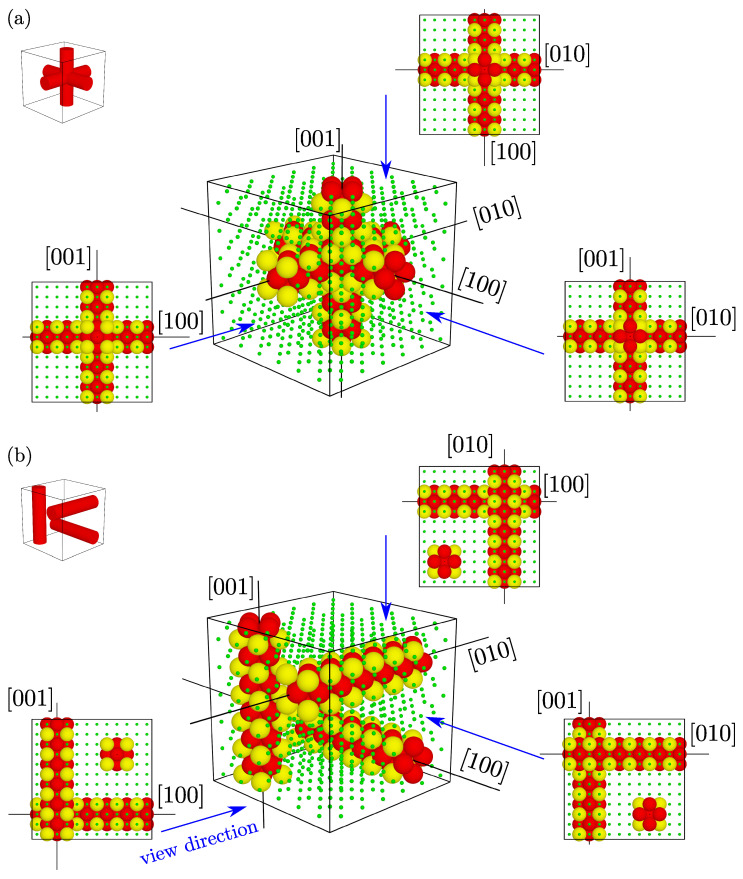
Illustration of the two different channel layouts (**a**) crossing nanochannels and (**b**) separate nanochannels. The included inserts present projections from the indicated directions for a precise presentation of the channel layouts. The yellow color has been used only to highlight the differences between the *D*-type and *S*-type channels. The green dots represent the matrix spheres located outside the nanochannels.

**Figure 3:**
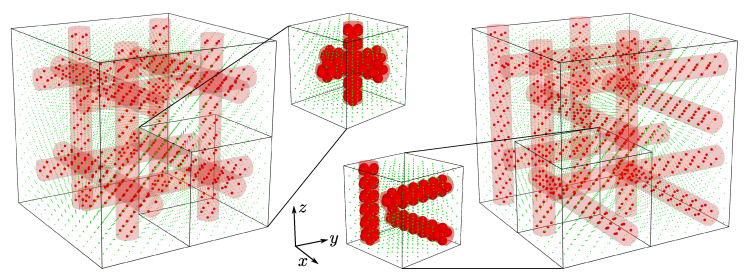
Illustration of different channel layouts studied on the basis of a 6×6×6 f.c.c. supercell periodically doubled in each direction. The left part of the image shows the layout with nanochannels crossing each other. The right part presents the layout with separate nanochannels. The radii of the nanochannels are equal to σ (*D*-type channels). The matrix particles (green spheres) were intentionally reduced in size to show the structure of the inclusions.

As mentioned above, the studied systems feature triple channel inclusions. The nanochannels are mutually orthogonal and oriented in [100], [010], and [001]-directions. There are several possible combinations as to how the three channels can be arranged in 3D space. In this work, we selected two border cases where (i) all the channels are crossing each other and (ii) the channels are separated by matrix particles and do not come into direct contact. In Figure 2, both channel layouts are presented, along with additional views for full information on different arrangement of nanochannels. Detailed data on inclusions in both layouts and sizes are given in Table 1. The layouts in the *D*-type variants have been illustrated in Figure 3, where they have additionally been doubled in each direction. The cylindrical translusive red shape marks the *D*-type nanochannel. After selected layouts of nanochannels are introduced into the f.c.c. model, the latter can be regarded as periodic repetitions of a single supercell. In the following part of this article, we will refer to the modification described simply as “the inclusion”.

The described models were studied under periodic boundary conditions. Results obtained for the periodic box containing the single supercell agreed, within the limit of an experimental error, with simulations of periodic box containing systems: doubled in one selected *x*-, *y*-, or *z*-direction (doubled supercell), doubled in any two directions (quadrupled supercell), and doubled in all three directions (octupled supercell) [50]. Thus, it was reasonable to simulate single supercells.

## 3. The Method

### 3.1. Theory

To calculate the elastic properties of the described models, computer simulations based on the idea of Parrinello and Rahman [62,63] were performed. The idea was implemented using the Monte Carlo (MC) method in the isobaric-isothermal ensemble (NpT) [58,64]. It allows one to calculate the complete elastic compliance tensor S of 21 elements from observations of shape fluctuations of a sample placed in the periodic box. All $S_{\alpha \beta \gamma \delta }$ elements are obtained from these shape fluctuations by calculating the strain tensor ε for the system under dimensionless pressure $p^{*}=p\beta \sigma^{3}$ as [58,63]:

(2) $$\varepsilon =\frac{1}{2}\left(h_{p}^{-1}.h.h.h_{p}^{-1}-I\right)\;,$$

where I is a unit matrix, h is a symmetric matrix formed by vectors defining the edges of a periodic parallelepiped, and $h_{p}$ is the reference matrix, i.e., the average value of the h matrix at equilibrium under dimensionless pressure $p^{*}$, $h_{p}\equiv \langle h\rangle$. It is worth noting that in the case of the systems studied in this work, the periodic box typically contains a unit supercell and the box matrix h defines its shape. The advantage of this approach is that it allows the unit cell to optimize the shape and size under arbitrary applied thermodynamic conditions. The symmetry of the h matrix allows one to avoid rotations of the system during simulation. In the next step, the elastic compliance tensor elements are related to the strain tensor components by the formula [58]:

(3) $$S_{\alpha \beta \gamma \delta }=\beta V_{p}\langle \Delta \varepsilon_{\alpha \beta }\Delta \varepsilon_{\gamma \delta }\rangle \;,$$

where $V_{p}=\left|\det\left(h_{p}\right)\right|$ is the volume of the system at the dimensionless pressure $p^{*}$, $\Delta \varepsilon_{\alpha \beta }=\varepsilon_{\alpha \beta }-\langle \varepsilon_{\alpha \beta }\rangle$, $\langle \varepsilon_{\alpha \beta }\rangle$ is the average in the NpT ensemble, and α,β,γ,δ = *x*, *y* or *z*. An expression for PR based on the knowledge of the elastic compliance tensor can be given in a general form [65]:

(4) $$\nu_{nm}=-\frac{m_{\alpha }m_{\beta }S_{\alpha \beta \gamma \delta }n_{\gamma }n_{\delta }}{n_{\zeta }n_{\eta }S_{\zeta \eta \kappa \lambda }n_{\kappa }n_{\lambda }}\;.$$

It can be seen from the above formula that the PR depends on the choice of two mutually orthogonal directions (represented as unit vectors): the one in which the external stress is applied (represented by the $\vec{n}$ vector), and the other in which PR is measured ($\vec{m}$). The Einstein summation convention is used on Greek indexes. For the sake of clarity, in the remaining part of the manuscript, we express the $S_{\alpha \beta \gamma \delta }$ tensor elements with the elastic compliance matrix $S_{ij}$ elements using the Voigt representation [52]. The Latin indices for the $S_{ij}$ elements of this symmetric square matrix take the values i,j=1,…,6. It should also be stressed that all calculations in this work concern infinitesimally small deformations (strains). In other cases, a different approach should be used, e.g., the one described in [4]. Such a case is outside the scope of this research. Further details on the applied method and calculations of the elastic properties are provided in previous articles [50,51].

### 3.2. Simulations

The research was carried out using numerical methods. The MC simulations were performed in the NpT ensemble. The size of the considered supercell matched 6×6×6 f.c.c. cells, thus containing N=864 spheres. The number of particles forming the inclusion varied depending on its size and layout, and is summarized in Table 1. The studied systems were subjected to dimensionless pressure $p^{*}=50$, 100, 250, and 1250. The values of $\sigma^{\prime }/\sigma$ ranged between 0.95 and (depending on the pressure) 1.1. Twenty five independent simulation runs were performed for each value of $\sigma^{\prime }/\sigma$ and $p^{*}$. Each simulation took at least $10^{7}$ MC cycles, from which the first $10^{6}$ was treated as the period in which the system reaches thermodynamic equilibrium and rejected from calculations. The remaining details of the computer simulations can be found in [51], and references therein.

## 4. Results and Discussion

Early studies showed that periodic arrays of nanochannel inclusions of particles with increased diameters, introduced in one of the principal crystallographic directions (e.g., [001]), substantially decrease the PR, thus improving the auxetic properties of such systems [49]. Later studies showed that, when nanochannels are combined with nanolayer inclusions (oriented orthogonally to the channel axis), increasing diameters of inclusion particles has the opposite effect. Such a hybrid inclusion completely removes the auxetic properties from the system [51]. This indicates that not only the size of the inclusion particles, but also the form of the inclusion, is one of the key factors influencing the elastic properties of the model. Moreover, such nanochannels, nanolayers, or their combination induced the change of the systems’ symmetry from cubic to tetragonal (422 symmetry class [52]). In this regard, it is interesting to test the changes exerted on elastic properties with a nanochannel inclusion designed to preserve the (effective) cubic symmetry of the system.

For this reason, we designed inclusions based on three nanochannels oriented along three main crystallographic directions: [100], [010], and [001]. There are several ways in which one can arrange three orthogonal nanochannels in space. We consider two border cases: (i) crossing nanochannels and (ii) separate nanochannels. Two sizes of the nanochannels were studied. All systems were subjected to four different values of external reduced pressure $p^{*}$.

**Figure 4:**
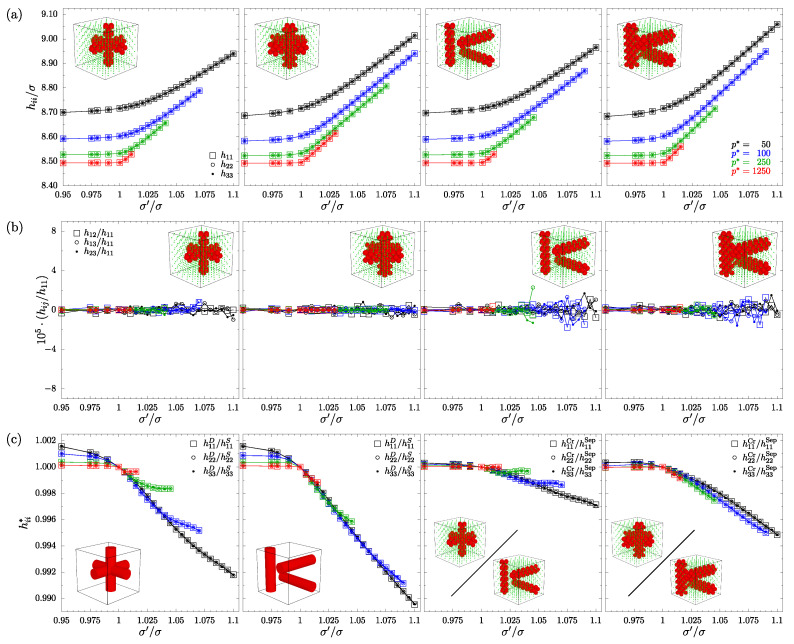
Box matrix components $h_{ij}$ for all the systems studied, plotted with respect to the scaling factor $\sigma^{\prime }/\sigma$ (row **a**), off-diagonal components divided by $h_{11}$ (row **b**). Row (**c**) presents the ratios of the diagonal components of the box matrix $h_{ii}^{*}=h_{ii}^{X}/h_{ii}^{Y}$ from the left: X,Y are *D*-type and *S*-type, respectively, for (i) crossing “Cr” and (ii) separate “Sep” nanochannels, and X,Y are crossing and separate nanochannels, respectively, for (iii) *D*-type and (iv) *S*-type systems. Data for different values of reduced external pressure $p^{*}$ are colored. In the case of figures (**a**,**c**), the simulation errors of the values are below 0.1% and the are considerably smaller than the symbols representing them. In the case of figures (**b**), the zero value is within the error bars.

In Figure 4, the data concerning the shapes of the studied systems are presented. Elements of the box matrix $h_{p}$ for all studied systems and pressures are plotted with respect to the ratio $\sigma^{\prime }/\sigma$ (the data corresponding to systems under different values of external pressure are indicated with different colors). The three diagonal components were plotted, with different symbols, on subfigures in row (a)—it can be seen that they all follow the same curve for the corresponding pressures. Apart from the fact that the volume of systems with separate channels is slightly higher (at most ≈0.5%) compared to the models with crossing channels (due to increased $N_{\mathrm{inc}}$), all the systems exhibit similar behavior—they preserve cubic shape. It is worth stressing that, in contrast to the crossing nanochannels, the separate nanochannel systems do not have cubic symmetry (due to the missing 4-fold symmetry axis). Thus, it was not obvious that they would preserve the perfectly cubic shape. The ratios of $h_{22}/h_{11}$ and $h_{33}/h_{11}$ are equal to 1 for all the cases studied, and the off-diagonal $h_{p}$ components are five orders of magnitude less than their diagonal counterparts, thus, considered zero (row (b) of Figure 4). Row (c) of Figure 4 presents the relations of $h_{ii}$ components between different studied system variants. Namely, (from the left) the relation between sizes of nanochannels in the same layouts, (i) crossing and (ii) separate nanochannels (first two plots) and the relations between the two layouts of the same size, (i) *D*-type and (ii) *S*-type systems (3rd and 4th plots). Subfigures corresponding to cases (i) and (ii) present expected behavior where, along with an increase of $\sigma^{\prime }/\sigma$, wider *S*-type nanochannels extend the systems to higher values of $h_{ii}$ than *D*-type systems (regardless of the channel layout). A similar effect is observed for cases (iii) and (iv), where the size of the systems with different channel layouts is compared for the same channel diameter. The separate nanochannel layout systems extend more due to the higher number of $N_{\mathrm{inc}}$ particles they contain. The differences in $h_{ii}$ grow along with the increase of channel diameter.

**Figure 5:**
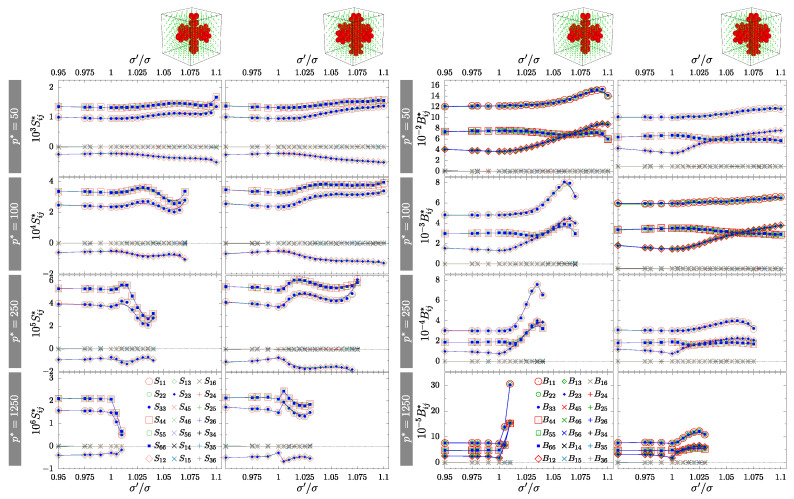
Components of the elastic compliance matrix S (**left**) and matrix of elastic constants B (**right**) for systems with crossing *D*-type and *S*-type nanochannels. Corresponding values of reduced external pressure $p^{*}$ are indicated in the figure. The simulation errors of the values are below 3% and they are considerably smaller than the symbols representing them. The quantities represented by cross and plus symbols (x and +) are equal to zero within the computational error.

**Figure 6:**
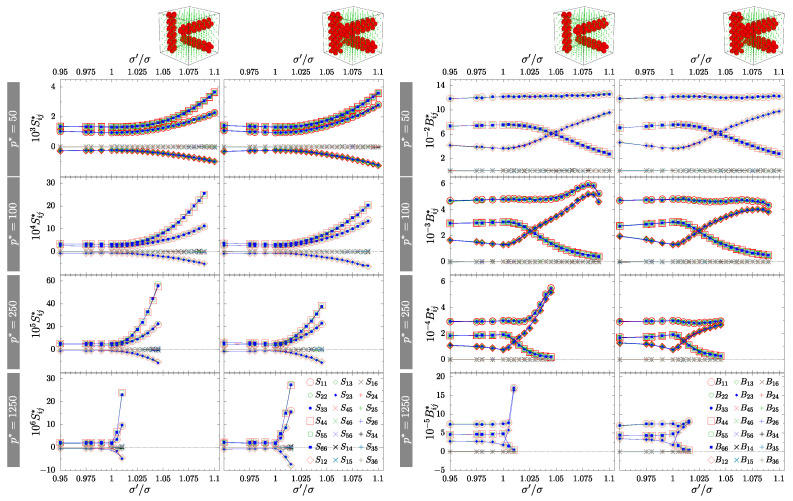
Components of the elastic compliance matrix S (**left**) and matrix of elastic constants B (**right**) for systems with separate *D*-type and *S*-type nanochannels. Corresponding values of reduced external pressure $p^{*}$ have been indicated in the figure. The simulation errors of the values are below 3% and they are considerably smaller than the symbols representing them. The quantities represented by cross and plus symbols (x and +) are equal to zero within the computational error.

To confirm the symmetry of the system, one has to examine the matrixes of the elastic compliance S or elastic constants B. The former were determined by the MC simulations, from the fluctuations of the shape of periodic box (h), while the latter are simply related to S (for details see Equation (7) in [58]). Both arepresented in Figure 5 and Figure 6 for crossing and separate channels, respectively. The values of S (left part) and B (right part)were organized in columns corresponding to different channel sizes *D*-type and *S*-type. Subfigures for increasing pressures were placed in descending rows. In both cases, the crossing nanochannels (Figure 5) and the separate nanochannels (Figure 6), we can see that all the required relations between matrix elements ($X_{11}=X_{22}=X_{33}$, $X_{44}=X_{55}=X_{66}$, $X_{12}=X_{13}=X_{23}$ and all the other elements equal to zero, where *X* stands for *S* or *B*) are met for both matrixes. Thus, all the studied systems with inclusions exhibit effective cubic symmetry. However, it should be stressed again that the systems with separate channels and with $\sigma^{\prime }/\sigma \neq 1$ are not cubic, due to the missing 4-fold symmetry axis.

One can see that in the case of separate channels (Figure 6) the values of elastic compliances increase substantially with the increase of $\sigma^{\prime }/\sigma$ (an increase of diameters of channel spheres). It is clear that this difference cannot be attributed to the difference in concentrations, *c*, between both layouts, as the differences in *c* are just too small. Moreover, the *c* value for *S*-type channels in the crossing layout is higher than the *c* value of *D*-type systems with separate channels (see Table 1).

The PR can be calculated based on either of the above (S or B) matrixes, but here we present the formulas for the PR for cubic symmetry expressed in terms of the $B_{11},\;B_{12}$ and $B_{44}$ elastic constants, for the main, isotropic ([100], [111]) [66]:

(5) $$\begin{array}{ccc} \nu_{\left[100\right]} & = & \frac{B_{12}}{B_{11}+B_{12}}\;, \end{array}$$

(6) $$\begin{array}{ccc} \nu_{\left[111\right]} & = & \frac{B_{11}+2B_{12}-2B_{44}}{2(B_{11}+2B_{12}+B_{44})}\;, \end{array}$$

and anisotropic ([110]) crystallographic directions [66]:

(7) $$\begin{array}{ccc} \nu_{\left[110\right]\left[1\bar{1}0\right]} & = & \frac{B_{11}^{2}-2B_{12}^{2}+B_{11}\left(B_{12}-2B_{44}\right)}{B_{11}^{2}-2B_{12}^{2}+B_{11}\left(B_{12}+2B_{44}\right)}\;, \end{array}$$

(8) $$\begin{array}{ccc} \nu_{\left[110][001\right]} & = & \frac{4B_{12}B_{44}}{B_{11}^{2}-2B_{12}^{2}+B_{11}\left(B_{12}+2B_{44}\right)}\;. \end{array}$$

**Figure 7:**
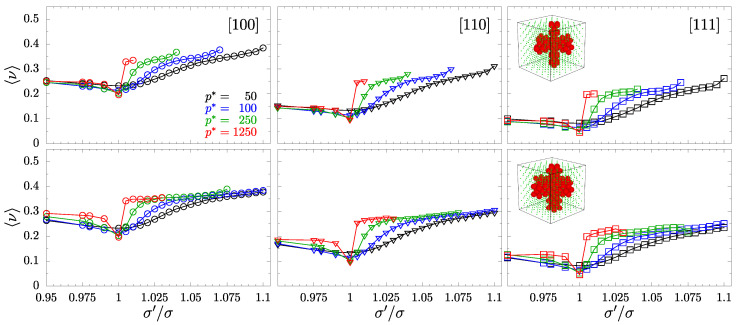
PR for selected $\vec{n}$-directions, indicated in the top right of their respective columns, averaged over all possible $\vec{m}$-directions. Different symbols (circles, triangles, and squares) correspond to PR in the directions [100], [110], and [111], respectively. The figure contains data for models with crossing nanochannels. Plots for the respective size of the nanochannel have been arranged in rows, as indicated by the miniature structure inserts. The values of the reduced external pressure $p^{*}$ have been indicated in colors.

**Figure 8:**
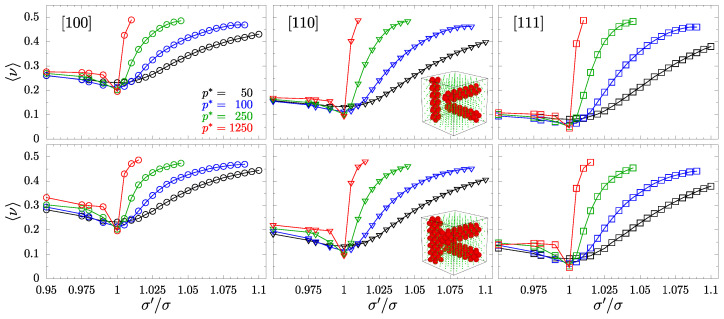
PR for selected $\vec{n}$-directions, indicated in the top left of their respective columns, averaged over all possible $\vec{m}$-directions. Different symbols (circles, triangles, and squares) correspond to PR in the direction [100], [110], and [111], respectively. The figure contains data for models with separate nanochannels. Plots for the respective size of the nanochannel have been arranged in rows, as indicated by the miniature structure inserts. The values of the reduced external pressure $p^{*}$ have been indicated in colors.

To examine the impact of inclusions on PR of the studied systems, we begin the analysis by plotting the averaged PR in selected (main) crystallographic directions, described by Equations (5) and (6), and the average of $\nu_{\left[110\right]}$. Figure 7 and Figure 8 (for crossing and separate nanochannel systems, respectively) present these PRs with $\vec{n}$-direction set as: [100], [110], and [111] (organized in respective columns). The values are averaged over all possible $\vec{m}$-directions, and arranged in rows corresponding to the respective nanochannel sizes, *D*-type (top) and *S*-type (bottom)—also indicated by the miniature structure inserts. One can see that changing the value of inclusion sphere diameters is always accompanied by an increase of the average PR. This increase is more dominant when $\sigma^{\prime }/\sigma >1$, especially in the case of separate nanochannels (Figure 8), where at higher pressures PR approaches 1/2 (the limit for 3D isotropic systems). The external pressure *p*, indicated in different colors, is also responsible for the increase of average PR, especially for separate channel layouts.

**Figure 9:**
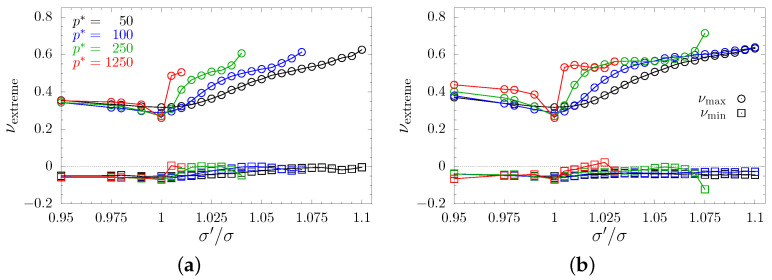
Extreme PR values for systems with (**a**) *D*-type and (**b**) *S*-type crossing nanochannels, plotted with respect to scaling factor $\sigma^{\prime }/\sigma$. Values for maximal and minimal PR have been marked with sphere and square symbols, respectively. Results obtained for different values of the reduced external pressure $p^{*}$ have been indicated in colors.

**Figure 10:**
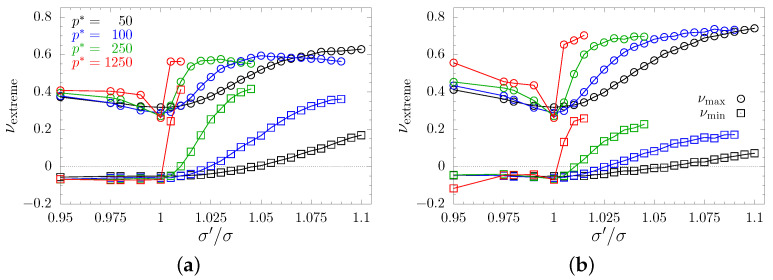
Extreme PR values for systems with (**a**) *D*-type and (**b**) *S*-type separate nanochannels, plotted with respect to scaling factor $\sigma^{\prime }/\sigma$. Values for maximal and minimal PR are marked with sphere and square symbols, respectively. Results obtained for different values of the reduced external pressure $p^{*}$ have been indicated in colors.

The average PR is good for an initial assessment of effects an inclusion exerted on the system. However, it does not provide the reader with the necessary insight into the changes in elastic properties. Thus, one should examine the changes in extreme values of PR caused by changing values of inclusions’ sphere diameters. Figure 9 and Figure 10 present maximal and minimal PRs found for any pair of ($\vec{n}$, $\vec{m}$)-directions in all the studied models. The maximal and minimal values are represented by circles and squares, respectively. As with previous plots, different values of the reduced external pressure $p^{*}$ are indicated by different colors. To find the global extreme of the PR, the studied systems were sampled in $10^{6}$ different $\vec{n}$-directions. As could be expected for cubic systems, presented extremes correspond to [110]-direction, or equivalent (e.g., $\left[1\bar{1}0\right]$, [101], [011], etc). One can see that typically maximal PR increases, especially with increasing values of the inclusion sphere diameters. With the exception of *S*-type nanochannels in separate layout (Figure 10b), the maximal PR reaches values around 0.6. A notable difference between channel layouts can be seen in minimal PR. In the case of crossing channels, the minimal (negative) PR increases only slightly and approaches 0, whereas in the case of separate nanochannels the minimal PR becomes positive. The increase is faster at higher pressures. It is worth noting that all possible PR values, at a given $\sigma^{\prime }/\sigma$, lie between the plotted curves for the given pressure. Thus, one can see that the models of separate *D*-type nanochannels exhibit the most narrow range of possible PR values. This range narrows along with an increase of $\sigma^{\prime }/\sigma$ and $p^{*}$. Another important note is that models with separate nanochannels effectively eliminate auxeticity from the system. This stands in contrast to single nanochannel inclusion that greatly enhances auxetic properties [49]. In the studied case of separate nanochannel systems, PR turns positive when $\sigma^{\prime }/\sigma >1.045$ ($\sigma^{\prime }/\sigma >1.055$ for *S*-type channels) under $p^{*}=50$. This threshold lowers along with the increasing pressure, and drops to $\sigma^{\prime }/\sigma >1.02$ ($\sigma^{\prime }/\sigma >1.025$ for *S*-type channels) under $p^{*}=100$ and to $\sigma^{\prime }/\sigma >1.01$ for $p^{*}=250$ (for both channel sizes). In the case of the highest studied value of pressure ($p^{*}=1250$), every studied system for $\sigma^{\prime }/\sigma >1$ is non-auxetic. A similar effect of canceling the auxetic properties of the system was observed earlier in hybrids of layer and channel inclusions [51], but the effect was also accompanied by the change of system’s symmetry from cubic to tetragonal. In the case of current, three-channel inclusion, we observe a complete lack of auxetic properties while preserving the effective cubic symmetry. Thus, cubic-like systems can be obtained without one of the characteristic features of most cubic systems [30], namely the negative value of PR in the $\left[110\right]\left[1\bar{1}0\right]$-direction.

Another thing to note is that for separate nanochannel systems, the values of average PR (presented in Figure 8) in the three presented directions are very close (for high values of $\sigma^{\prime }/\sigma$). One might have the impression that the value of averaged PR does not depend on the choice of the (loading) $\vec{n}$-direction, meaning that the system becomes (on average) elastically isotropic. To verify this, we must examine whether the relation required for isotropic systems if fulfilled:

(9) $$B_{44}=\frac{1}{2}\left(B_{11}-B_{12}\right).$$

**Figure 11:**
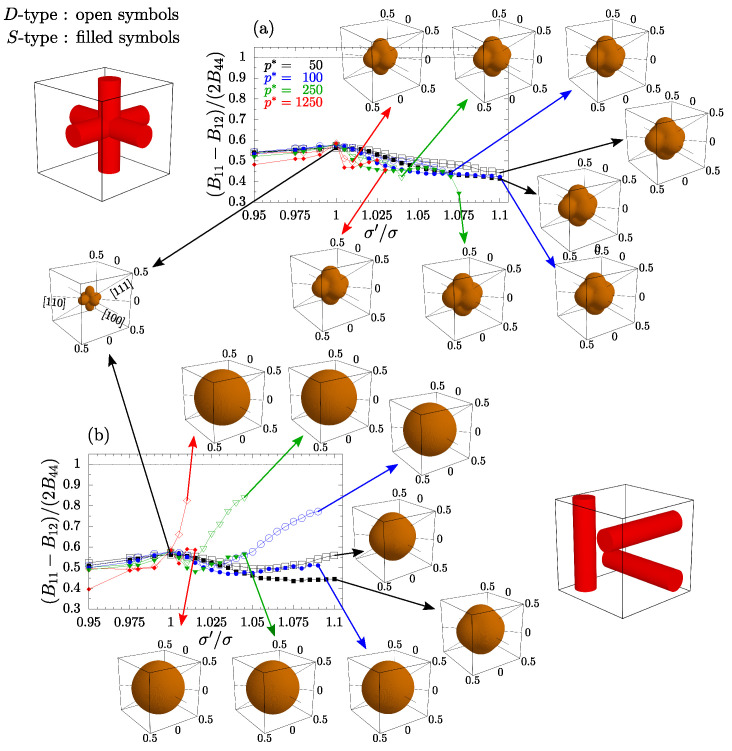
Calculated relative isotropy criterion (Equation (9)) for studied systems with (**a**) crossing and (**b**) separate nanochannel inclusions. The inserts present the average PR, plotted in the spherical coordinate system, for, respectively, the marked values of $\sigma^{\prime }/\sigma$ (corresponding to the maximal studied values of $\sigma^{\prime }/\sigma$), for all systems studied and under all pressures (indicated in colors). The open and closed symbols correspond to the *D*-type and *S*-type systems, respectively.

Figure 11 shows that the impact on the anisotropy of the system is qualitatively different for both crossing (Figure 11a) and separate (Figure 11b) inclusion layouts. The plots indicate that the crossing nanochannel systems are less isotropic when $\sigma^{\prime }/\sigma \neq 1$. In the case of separate nanochannels, numerical data show that increasing the diameters of channel particles significantly reduces the anisotropy of the *D*-type system (Figure 11b). However, the inserts in Figure 11, which present the average PR plotted in spherical coordinate system for the highest presented values of $\sigma^{\prime }/\sigma$, for each system and each pressure, show that even for *S*-type channels in the separate channel layout an average PR does not depend (considerably) on $\vec{n}$-direction, especially at higher pressures. It can be seen that it is almost a perfect sphere for the case of separate nanochannel systems at $p^{*}=250$ and 1250, whereas the crossing nanochannel systems exhibit anisotropic behavior, characteristic to monodisperse systems with hard spheres.

**Figure 12:**
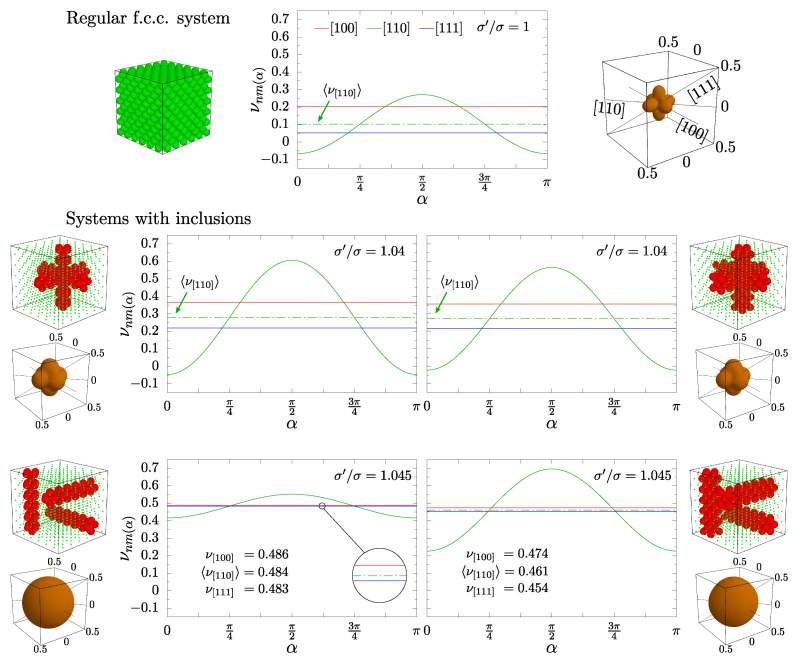
PR as a function of an angle α (designating $\vec{m}$-direction), plotted when loading is applied in the main crystallographic directions (indicated in different colors) for the systems studied at a reduced external pressure $p^{*}=250$ and common maximal $\sigma^{\prime }/\sigma$ values for *D*-type and *S*-type systems, respectively. The values can be easily compared to a monodisperse system without inclusions (at the top).

However, it should be noted that Figure 11 can be misleading. One could expect that systems with anisotropy parameters closer to 1 are more isotropic. As one can see, this is not the case for separate *S*-type nanochannel systems. The reason for the difference in isotropy of different systems is explained in Figure 12. PR has been plotted as a function of the $\vec{m}$-direction (here parametrized by an angle α), for the three previously discussed cases of the applied strain directions. The data are presented for systems at pressure $p^{*}=250$ and the highest $\sigma^{\prime }/\sigma$ value that is common both *D*-type and *S*-type systems, respectively, for a given layout. As expected, for cubic symmetry, the directions [111] and [100] are isotropic (the PR value does not depend on α), whereas the [110]-direction depends on the choice of the measurement direction ($\vec{m}$). The presented values can be easily calculated based on the knowledge of the elastic constants $B_{11},\;B_{12}$ and $B_{44}$, using Equations (5)–(8). The last two formulas correspond to minimal and maximal values of the $\nu_{\left[110\right]}$ curves, respectively, in Figure 12. One can see that the data for crossing channels are qualitatively the same as in the case of regular, monodisperse system. Introduced *D*-type and *S*-type inclusions merely increased the values of presented PRs and changed the amplitude of $\nu_{\left[110\right]}$, compared to the system without inclusions. On the other hand, one can see that the average PR in the [110]-direction for the separate nanochannel system is very close to the remaining two, as opposed to the pristine cubic and crossing nanochannel systems. In the case of the *D*-type system it differs only by 0.4%. For the presented case, the values of the average $\nu_{nm}$ are equal to 0.486, 0.484 and 0.483 for [100], [110], and [111] directions, respectively. It would seem that PR could be considered to be an indicator of the system’s anisotropy. Figure 11 and Figure 12 show that this is not the case for the average PR. The 3D plots show almost identical sphere-like shapes obtained for systems with anisotropy parameter equal to 0.4 and 0.8.

**Figure 13:**
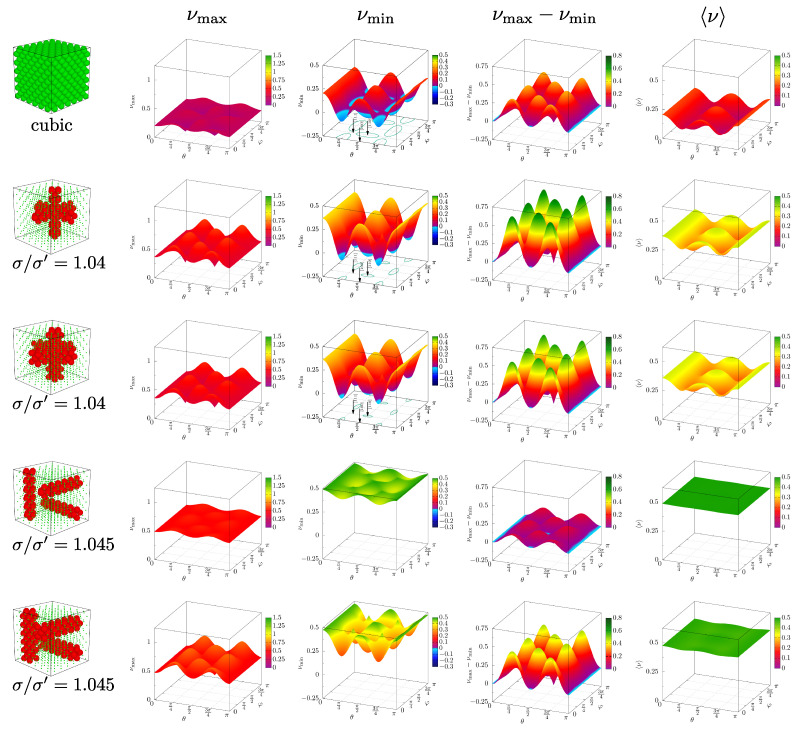
Surfaces composed of: maximal, minimal, their difference and the average PR (in respective columns) as a function of $\vec{n}$-direction expressed by the polar and azimuth angles (θ, φ), presented for the cubic system and all the inclusion variants studied. The cases presented correspond to the data in Figure 12, for pressure $p^{*}=250$.

To further aid in the visualization of the elastic properties of studied models, the data from Figure 12 have been extended to include more than the three main crystallographic directions. Figure 13 presents data for systems at $p^{*}=250$, namely the minimum and maximum PR in $5\times 10^{4}$ different $\vec{n}$-directions presented in the form of 3D surfaces with respect to polar and azimuth angles θ, φ. The directions presented in the previous figures are marked with arrows pointing at the corresponding pairs of θ, φ angles. The data for the respective systems is organized in rows, starting from the monodisperse system, followed by *D*-type and *S*-type systems for crossing and separate layouts. The columns contain surfaces of maximal, minimal, their difference, and the average PR. The contours on the horizontal θ-φ plane indicate the pairs of angles, for which PR is negative. It can be seen that for separate channels (the two bottom rows) the average PR is qualitatively different than for crossing channels. In the former case, despite the differences in extreme values, the average PR changes only by a small amount between directions. For *D*-type channels, it is almost a flat surface. However, it can be seen that differences between maximal and minimal PR are not small.

## 5. Conclusions

It was shown that even small modifications of the crystal structure can exert a considerable impact on the macroscopic properties of the system. The inclusion of hard particles with only a few percent difference in their diameters can significantly modify their elastic properties. The two layouts of inclusions composed of identical nanochannels resulted in substantially different elastic behavior in the final systems. This indicates that besides the properties of particles forming the inclusions, their shape, size, and orientation also have a key influence on elastic properties of the model material. It was found that periodic arrays of three nanochannels, oriented orthogonally to each other, either crossing or remaining separate, cause the overall increase of PRs. This unexpected result (keeping in mind that similar arrays composed of a single nanochannel greatly enhance auxetic properties) shows how difficult it is to predict the macroscopic impact of such microscopic modifications. It is worth noting that the studied three-channel inclusions were preserved the cubic shape of the simulated samples, which exhibit effective cubic symmetry (described by only three independent elastic constants). The different impact of inclusion layouts is also reflected in the anisotropy of the models. In the case of the separate *D*-type nanochannel layouts, the systems are effectively more isotropic at higher pressures and higher values of diameters of inclusion spheres. However, *S*-type nanochannels in the same layout also show only little changes of the average PRs in different directions.

This article presents the potential of structural modifications as a tool for altering the macroscopic elastic properties of materials. With a better understanding of how microscopic modifications to the crystalline structure influence its macroscopic elastic properties, it should be possible to design systems with tailored elastic properties and PR to given applications. One of the ways to reach this understanding is to perform extensive simulations of model systems. In particular, for systems with cubic symmetry, studies of the impact of channels on the elastic properties should include other diameters of the channels, various distances between their axes, and their different orientations.

We hope that the results presented in this article will constitute a starting point for real experiments in the areas of material engineering and metamaterials.

## Figures and Tables

**Table 1:** Detailed number of particles in nanochannels of different sizes and layouts. Differences between layouts are due to the number of shared particles in the crossing channels. The values vary with the total number of particles. The values presented are for the system of N=864 particles, which corresponds to 6×6×6 f.c.c. cells.

		Crossing Channels		Separate Channels	
**Label**	**Diameter [** σ **]**	$N_{\mathrm{inc}}$	c **[%]**	$N_{\mathrm{inc}}$	c **[%]**
*D*-type	2	76	8.8	90	10.42
*S*-type	$2\sqrt{2}$	136	15.74	162	18.75

## Data Availability

The data presented in this study are available on request from the corresponding author (J.W.N.).

## Abbreviations

The following abbreviations are used in this manuscript (listed in order of occurrence in the text):

PR	Poisson’s ratio
f.c.c.	face centered cubic
HS	Hard Sphere (potential)
MC	Monte Carlo
